# Exclusive breastfeeding rate and related factors among mothers within maternal health WeChat groups in Jiaxing, Zhejiang province, China: a cross-sectional survey

**DOI:** 10.1186/s13006-022-00521-5

**Published:** 2022-11-24

**Authors:** Chun-Yan Fu, Xue-Juan Tang, Ling-Pei Pan, Hai-Ying Jin, Juan-Feng Yao, Li-Zhong Wang

**Affiliations:** 1 Department of Maternal Health, Jiaxing Maternity and Children Health Care Hospital, Affiliated Women and Children Hospital, Jiaxing University, Jiaxing, Zhejiang Province China; 2 Department of Maternal Health, Jiaxing Xiuzhou Maternity and Children Health Care Hospital, Jiaxing, Zhejiang Province China; 3 Department of Public Health, Jiashan Maternity and Children Health Care Hospital, Jiaxing, Zhejiang Province China; 4 Department of Maternal Health, Tongxiang Maternity and Children Health Care Hospital, Jiaxing, Zhejiang Province China; 5 Department of Anesthesiology, Jiaxing Maternity and Children Health Care Hospital, Affiliated Women and Children Hospital, Jiaxing University, Jiaxing, Zhejiang Province China

**Keywords:** Cross-sectional study, Exclusive breastfeeding, Maternal health care, WeChat application

## Abstract

**Background:**

The health workers in Jiaxing of China have established maternal health WeChat groups for maternal health education and management since 2019. Pregnant women in Jiaxing are invited to join the WeChat groups and a health worker as the group manager provides health education and individual counselling for women within the group. This study aimed to investigate the exclusive breastfeeding (EBF) status up to six months and its associated factors among the mothers of infants aged 7-12 months within the WeChat groups.

**Methods:**

This was a cross-sectional survey on healthy mothers with infants aged 7-12 months from seven maternal health WeChat groups in October 2021 in Jiaxing, China. EBF was defined as breastfeeding infants exclusively up to six months. Data including breastfeeding practice from birth to six months, maternal sociodemographic and obstetric characteristics, hospitalization information, work related factors and reasons for non-EBF up to six months were collected using an online self-administered questionnaire. A multivariable logistic regression analysis was performed to identify the factors independently associated with EBF up to 6 months.

**Results:**

A total of 822 mothers were included in this study. Among them, 586 mothers (71.3%) exclusively breastfed infants up to six months. Multivariable logistic regression analysis showed that older maternal age (adjusted odds ratio [AOR] 0.956; 95% confidence interval [CI] 0.917, 0.997) and perceived insufficient breast milk (AOR 0.104; 95% CI 0.072, 0.149) were associated with lower odds of EBF up to six months. The five of common reasons for non-EBF up to six months were no or insufficient breast milk (59.8%), return to work (23.9%), no flexible nursing breaks at work (18.2 %), infant crying or feeling tired or troubled with breastfeeding (9.7%), and nipple and breast problems (9.3%).

**Conclusion:**

About 71.3% of infants were exclusively breastfed until six months of age in our WeChat groups. Perceived insufficient breast milk and work related factors are the main barriers to EBF up to six months in this setting. However, further comparative study is needed to confirm the effect of WeChat groups on breastfeeding.

## Background

World Health Organization (WHO) has set one of the global nutrition target as increasing the exclusive breastfeeding (EBF) rates up to 50% by 2025 [1]. In China, the EBF rates, as the measurement of exclusively breastfeeding infants under six months during the past 24 hours, were 20.7% in 2013 [2] and 29.5% in 2018 [3] as estimated by the nationally representative surveys. Hence, how to improve EBF rates has become one of the main challenges for public health care in China. There are many factors that can affect breastfeeding, including historical, socioeconomic, cultural, and individual factors; among them, health education and support provided by health professionals play an important role in breastfeeding outcomes [4–9].

WeChat, the most widely used mobile communication application in mainland China, supports one-on-one texting, group chats, multimedia sharing and more. To improve maternal health education and management, the health workers in Jiaxing, a relatively developed medium-sized city on the east coast of China, have established dedicated maternal health WeChat groups as part of traditional maternal health care since 2019. Jiaxing consists of five counties and two districts with a population of 5 400 868 and 37 366 births in 2020. Pregnant women in Jiaxing area are invited to join the WeChat groups on a voluntary basis when they attend the first antenatal clinics (about 4 months prenatal). Currently, each county and district in Jiaxing has established its own maternal health WeChat group, leading to a total of seven WeChat groups with women accounting for about 15% of the overall pregnant women in Jiaxing area. Each WeChat group has approximately 200-500 women covering the period from about 4 months prenatal to at least 12 months after birth, and a health worker as the group manager who provides weekly group education and individual counselling at any time on maternal health care including breastfeeding education and support. Also women within the group can share their breastfeeding experiences with each other through text and/or audio messages, as this is one of the WeChat group functions and encouraged by the group managers. The aim of this cross-sectional study was to investigate the prevalence of EBF from birth to six months among the mothers of infants aged 7-12 months within our seven maternal health WeChat groups. Additionally, the related factors potentially affecting EBF up to six months in this setting were also assessed.

## Methods

### Study design

This is a cross-sectional study which was conducted using an online self-administered questionnaire in October 2021 in Jiaxing, Zhejiang province, China. The inclusion criteria were healthy mothers with infants aged 7-12 months in our seven maternal health WeChat groups. The exclusion criteria were multiple gestations, premature delivery before 34 weeks, mastitis or prior breast surgery, major fetal congenital malformations, or any other problems that could affect breastfeeding. The study is reported according to the Strengthening the Reporting of Observational Studies in Epidemiology (STROBE) guidelines [10].

### Data collection

The questionnaire was designed by the researchers based on previous studies [3, 7] to address the survey objectives. It comprised a total of 28 items by using dichotomous, multiple-choice or open-ended questions, including breastfeeding practice from birth to six months, maternal sociodemographic characteristics, knowledge about the general breastfeeding recommendations by WHO (i.e. optimal infant breastfeeding should be initiated within the first hour of birth, EBF continue for six months and then appropriate complementary feeding should commence together with breastfeeding for at least two years) [11], obstetric information, breastfeeding support during hospitalization, and work related factors (Table 1). Regarding skin-to-skin contact and breastfeeding initiation after birth, the questionnaire asked participants if skin-to-skin contact started within 10 minutes (immediately) and breastfeeding started within the first hour after birth, respectively. To avoid recall bias, WHO recommends the indicator definition of EBF under six months is the percentage of infants 0-5 months of age who were fed exclusively with breast milk during the previous day [12]. Because this study only enrolled the mothers with infants aged 7-12 months, we investigated the breastfeeding practice from birth to six months. There were three options for the breastfeeding practice up to six months (i.e. EBF, mixed feeding or no breastfeeding). We explained in detail to the participants that EBF meant that they fed infants exclusively with breast milk without any additional food or drink, except for medicines, vitamins, and minerals from birth to six months. Participants who chose “mixed feeding” or “no breastfeeding” were grouped into non-EBF group and were asked to further report the reasons for non-EBF (open-ended and can be multiple).

**Table 1:** Type and contents of questions in the self-administered questionnaire

Type	Contents
Dichotomous	Parity, marital status, resident area, household register, knowledge about the breastfeeding recommendations, pregnancy and postnatal complications, baby gender, skin-to-skin contact within 10 minutes after birth, rooming-in, breastfeeding initiation within the first hour after birth, EBF during hospitalization, perception of insufficient breast milk, paid maternity leave, breastfeeding room at workplace
Multiple-choice	Breastfeeding practice from birth to six months (EBF^a^, mixed feeding or no breastfeeding), educational level, employment status, monthly family incomes, mode of delivery,
Open-ended	Mother age, mother height, mother weight, birth weight, ethnic origin, gestational age, reasons for non EBF

*Abbreviations*: *EBF* exclusive breastfeeding

^a^EBF was defined as breastfeeding exclusively for infants from birth to six months

The questionnaire was designed as simple as possible for participants to easily understand and make choices. Prior to the formal release, the questionnaire was pretested among 10 unselected mothers who did not included in the final survey to evaluate the appropriate wording and acceptability. Then the questionnaire was released online in the seven WeChat groups and all eligible mothers were invited to this survey. Each WeChat group manager who had been trained specifically for this survey was responsible for giving a full explanation to potential participants about the purpose of the survey, eligibility and exclusion criteria, questionnaire questions, and for answering any questions raised by the participants during the study via in-group text and/or audio messages. They also explained to the participants the voluntary and anonymity nature of the study including their right to not to participate in or to withdraw from the study at any time, but once they submitted the questionnaire meant they agreed to participate in the study. If agreed, participates were asked to complete and submit the questionnaire online on their mobile phones within two weeks.

### Ethical considerations

This study was approved by the Local Research Ethics Committee of Jiaxing Maternity and Children Health Care Hospital (Approval No. 2021-F-61). It was a voluntary and anonymous online survey, participants were informed that completing and submitting the questionnaire meant they agreed to participate in the study. As such, the requirement for written informed consent was waived by the Ethics Committee.

### Data analysis

The primary outcome was the self-reported EBF status from birth to six months. Continuous variables were presented as median (range), categorical variables were presented as number and percentage. Normal distribution for continuous variables was checked using the Shapiro-Wilk test and visual plot inspection. As the continuous variables were not normally distributed, univariate analysis was performed using Mann-Whitney *U* test, or the *x* ^2^ test and Fisher’s exact test to compare the differences between mothers with and without EBF. A multivariable logistic regression model was used to determine the different independent variables associated with EBF status up to six months by the adjusted odds ratio (AOR) and 95% confidence interval (CI). The independent variables that entered into the regression model were those of being significant in univariate analysis (*P*<0.10) or of possibly influencing EBF indicated by previous studies. Multicollinearity was assessed using the tolerance and variance inflation factor, with tolerance > 0.10 and variance inflation factor < 10.0 were considered acceptable. We did not perform a formal sample size calculation, but used a convenience sampling method. Data were analyzed using SPSS version 19.0 for Windows (IBM Corp., Armonk, NY, USA). A two-sided *P* < 0.05 was considered as statistically significant.

## Results

### Sample characteristics

Based on the estimates of the group managers, there were about 1,140 eligible mothers in the seven WeChat groups at the survey time. A total of 857 mothers online completed and submitted the questionnaire (response rate was 75.2%). Thirty-five mothers were excluded due to multiple gestations (*n*=27), premature delivery before 34 weeks (*n*=5), postpartum severe hemorrhage (*n*=2) or infections (*n*=1), leaving 822 mothers included in this study. There were no significant differences in background characteristics between excluded and included mothers (data not shown). All submitted questionnaires were complete.

The median age of participants was 29.6 (range 17-47) years. All participants were of Han ethnic and received a junior high school education or above (i.e. all had completed at least the nine-year compulsory education). The majority of surveyed mothers were married (98.1%), employed full-time or part-time (86.5 %) with a monthly family income of more than 5 000 RMB (91.1%), and aware of all three WHO recommendations about breastfeeding (86.1%). All mothers gave birth at seven public hospitals located in Jiaxing, of which five had Baby-Friendly Hospital Initiative (BFHI) certificates. With respect to obstetric and hospitalization information, 89.9% of mothers reported term (> 37 weeks), 67.3% reported primiparous, 65.3% reported vaginal delivery, 92.5% reported room-in, 88.6% reported skin-to-skin contact with their infants within 10 minutes after birth, 67.2% reported breastfeeding initiation within the first hour of birth and 88.9% reported EBF during hospitalization. In addition, of the full-time employed mothers (*n*=480), the majority (87.5%) reported that they returned to work after a paid maternity leave of 128 days for vaginal delivery and 143 days for cesarean delivery, but 72.7% reported that there was no independent breastfeeding room at their workplaces.

### EBF status up to six months and associated factors

Overall, 586 mothers reported that they exclusively breastfed their infants until six months of age, with a rate of EBF up to six months of 71.3% (95% CI 68.2, 74.4%). Univariate analysis showed that higher odds of EBF up to six months were found among the mothers with the following characteristics: younger (*P*=0.001), lower body mass index (BMI) (*P*=0.015), unemployed (*P*=0.003), vaginal delivery (*P*=0.014), breastfeeding initiation within the first hour post birth (*P*=0.017), and no perception of insufficient breast milk (*P*<0.001) (Table 2). After adjustment for other variables, multivariable regression analysis indicated that only older maternal age (AOR 0.956; 95% CI 0.917, 0.997) and perceived insufficient breast milk (AOR 0.104; 95% CI 0.072, 0.149) were associated with lower odds of EBF up to six months compared to the counterparts (Table 3).

**Table 2:** Characteristics of study sample by EBF status up to six months

Variables	EBF (*n*=586)	Non-EBF (*n*=236)	*P*-value^a^
Maternal age, years	29 (17-43)	30 (21-47)	0.001
BMI, kg·m^-2^	21.5 (15.6-38.5)	22.1 (15.8-35.2)	0.015
Household registration			
Local	411 (70.1)	176 (74.6)	0.202
Non-local	175 (29.9)	60 (25.4)	
Resident area			
Urban	348 (59.4)	147 (62.3)	0.442
Rural	238 (40.6)	89 (37.7)	
Marital status			
Married	572 (97.6)	234 (99.2)	0.174
Unmarried	14 (2.4)	2 (0.8)	
Educational level			
Junior high school or below	87 (14.8)	29 (12.3)	0.374
High school	101 (17.2)	35 (14.8)	
College or above	398 (67.9)	172 (72.9)	
Employment status			
Unemployed	83 (14.2)	28 (11.9)	0.003
Part-time	182 (31.1)	49 (20.8)	
Full-time	321 (54.8)	159 (67.4)	
Monthly family incomes, RMB			
<5 000	49 (8.4)	24 (10.2)	
5 000-10 000	244 (41.6)	97 (41.1)	0.057
10 001-19 999	208 (35.5)	66 (28)	
≥ 20 000	85 (14.5)	49 (20.8)	
Gestational age			
34- 37 weeks	61 (10.4)	22 (9.3)	
> 37 weeks	525 (89.6)	214 (90.7)	0.640
Parity			
Primiparas	399 (68.1)	154 (65.3)	0.433
Multiparas	187 (31.9)	82 (34.7)	
Pregnancy complications			
Yes	46 (7.8)	21 (8.9)	0.619
No	540 (92.2)	215 (91.1)	
Postnatal complications			
Yes	9 (1.5)	5 (2.1)	0.558
No	577 (98.5)	231 (97.9)	
Mode of delivery			
Vaginal delivery	398 (67.9)	139 (58.9)	0.014
Cesarean delivery	188 (32.1)	97 (41.1)	
Birth weight, g			
≤ 2500	21 (3.6)	13 (5.5)	0.210
>2500	565 (96.4)	223 (94.5)	
Baby sex			
Male	309 (52.7)	122 (54.7)	0.788
Female	277 (47.3)	114 (48.3)	
Rooming-in			
Yes	544 (92.8)	216 (91.5)	0.521
No	42 (7.2)	20 (8.5)	
Breastfeeding initiation after birth			
≤ 1h	408 (69.6)	144 (61.0)	0.017
>1h	178 (30.4)	92 (39.0)	
Skin-to-skin contact within 10 minutes after birth			
Yes	527 (89.9)	201 (85.2)	0.052
No	59 (10.1)	35 (14.8)	
Knowledge about breastfeeding recommendations^b^			
Yes	511 (87.2)	197 (83.5)	0.162
No	75 (12.8)	39 (16.5)	
Perception of insufficient breast milk			
Yes	151 (25.8)	182 (77.1)	<0.001
No	435 (74.2)	54 (22.9)	
Breastfeeding room at workplace^c^			
Yes	92 (28.7)	39 (24.5)	0.339
No	229 (71.3)	120 (75.5)	

Data are presented as median (range) or number (percentage)

*Abbreviations*: *BMI* body mass index, *EBF* exclusively breastfeeding

^a^*P*-value was based on Mann-Whitney *U* test for non-normally distributed continuous variables, and the *x*^2^ test or Fisher’s exact test for categorical variables

^b^“Yes” meant knowing all three WHO recommendations

^c^From the full-time employed mothers (*n*=321 in EBF, *n*=159 in non-EBF)

**Table 3:** Multivariable logistic regression of the associated factors with EBF status up to six months

Variables	OR (95% CI)	AOR (95% CI)
Maternal age	0.935 (0.903, 0.969)	0.956 (0.917, 0.997)
Perceived insufficient breast milk	0.103 (0.072, 0.147)	0.104 (0.072, 0.149)

*Abbreviations*: *AOR* adjusted odds ratio, *CI* confidence interval, *EBF* exclusively breastfeeding, *OR* odds ratio

### Reasons for non-EBF up to six months

In 236 non-EBF mothers, the top five reasons given for non-EBF up to six months were no or insufficient breast milk (*n*=141, 59.8%), inability to breastfeed their infants as needed after return to work (*n*=56, 23.9%), no flexible nursing breaks at work (*n*=43, 18.2 %), infant crying or feeling tired or troubled with breastfeeding (*n*=23, 9.7%), and nipple and breast problems (*n*=22, 9.3%), respectively. Other reasons included the concern that breast milk alone was not sufficient for infant’s nutritional needs (*n*=17, 7.2%), infant’s weight below the standard (*n*=16, 6.8%), no breastfeeding room or refrigerator for expressing or storing breast milk at workplace (*n*=16, 6.8%), perceived inconveniences or discomfort of breastfeeding in public (*n*=10, 4.2%), maternal illness (*n*=6, 2.5%), and pain or discomfort (*n*=4, 1.7%).

## Discussion

The current study reveals that the EBF rate, as the measurement of exclusively breastfed infants from birth to six months, is 71.3% among the mothers of infants aged 7-12 months within our maternal health WeChat groups. However, considering that the participants were only from the WeChat groups, further comparative study is needed to confirm the role of WeChat groups in promoting breastfeeding.

Health workers have played an important role in breastfeeding initiation and continuation [4–9]. In the traditional model of maternal health care, women are required to attend antenatal and postnatal clinics or classes regularly, and health workers are needed to follow up the mothers by telephone calls or home visits after their hospital discharge. This could decrease the compliance of women and increase the workload of health workers, which in turn diminishes the effectiveness of maternal health care. Because WeChat is now universally used by almost all women of childbearing age in our area, we adopt the establishment of maternal health WeChat groups as part of routine maternal health care. To facilitate management, we have established seven WeChat groups according to the administrative regions, namely, one WeChat group is responsible for the perinatal women of a county or district. In this way, health workers can provide regular breastfeeding education for all women and individual counseling for those who are experiencing feeding problems. The higher awareness rate (86.1%) of all three breastfeeding WHO recommendations in this study also reflects the effectiveness of this model of health education. In addition, the mothers within the group can also share their breastfeeding experiences with each other, thereby enhancing their breastfeeding confidence.

We acknowledge that other factors could also contribute to this higher rate of EBF up to six months in this study. In particular, most mothers gave birth at the BFHI certificate hospitals which adopted the “Ten Steps to Successful Breastfeeding” launched by WHO [13]. Therefore, it is not surprising that a higher proportion of mothers, even for those with cesarean delivery, reported that they had skin-to-skin contact immediately after birth, rooming-in with their infants, breastfeeding initiation within the first hour and EBF during hospitalization in our study. These interventions have all been shown to be strong contributors to establishment and continuation of breastfeeding [7, 14–17].

In line with previous studies [4, 5, 18–20], we found that there were significant differences in maternal age, BMI, employment status, mode of delivery, breastfeeding initiation, and perception of insufficient breast milk between mothers with and without EBF based on the univariate analyses. However, multivariable regression analysis showed that only maternal age and perception of insufficient breast milk were statistically associated with EBF up to six months, especially that perceived insufficient breast milk significantly decreased the odds of EBF from birth to six months. Regarding the relationship between maternal age and EBF, data in the literatures are conflicting. Some studies observed that older mothers were associated with lower EBF compared to younger ones [3, 5]; in contrast, other studies showed that older mothers were more likely to practice EBF than younger ones [19, 21]. In this study, maternal age was found to be negatively but marginally associated with EBF, indicating a limited effect.

The lack of associations of employment status and breastfeeding initiation with EBF up to six months may be due to the fact that the majority of surveyed mothers were employed and had breastfeeding initiation within the first hour. As for mode of delivery, previous studies suggested that women with cesarean delivery were less likely to EBF than those with vaginal delivery [3, 14]. Delayed onset of lactation, disrupted mother-infant interaction, inhibited infant suckling and poor pain relief may mediate the effects of caesarean delivery on breastfeeding [22]. In the current study, although caesarean delivery was less common in EBF mothers than in non-EBF mothers, it was not statistically associated with EBF up to six months after adjustment for other confounding variables. This result is consistent with the study by Ruan et al [5], and suggests that if the mothers receive adequate breastfeeding support during hospitalization, caesarean delivery is not necessarily a barrier to EBF. This may be especially important considering that a relatively higher caesarean delivery rate in China [23]. In addition, we did not found the differences in mothers’ educational level, monthly family incomes, skin-to-skin contact, and room-in between mothers with and without EBF, those have been identified as the factors related to EBF in previous studies [2, 3, 8, 17]. Again, sample characteristics and hospital practices may explain the discrepancies between this study and other studies.

With regard to the reasons for non-EBF up to six months, no or insufficient breast milk is the foremost one reported by the non-EBF mothers. This is consistent with the results of multivariable regression analysis and of other studies [3, 5, 6]. However, in fact, only few mothers have physiological insufficient milk supply and most mothers can produce enough breastmilk to meet their infant’s demand [24]. As such, this result may imply the inadequate education and guidance provided by health workers on this issue. The next main reasons are those work related factors, including inability to breastfeed their infants as needed after return to work and lack of flexible breaks at work. Notably, although fewer mothers (6.8%) stated no breastfeeding room or refrigerator at workplaces as the reason for non-EBF, 72.7% of the employed mothers reported there was no breastfeeding room at their workplaces. Other reasons are various, including infant crying or mother feeling tired or troubled with breastfeeding, nipple and breast problems, the concern about breast milk alone being not sufficient for infant’s needs, and perceived inconveniences or discomfort of breastfeeding in public. Fortunately, most reasons listed above can be amended through education and interventions. For example, health workers can guide mothers how to tell the difference between physiological and perceived insufficient breast milk, prepare mothers for tiredness and fatigue, improve mothers’ ability to soothe their infants, and eliminate their concern about insufficient breast milk nutrition. Returning to work before six months is still the common reason of early weaning breastfeeding for working mothers [5, 7, 9]. Thus, breastfeeding-friendly work policies and environments are needed for improving EBF among those mothers. For example, a relatively long maternity leave can extend breastfeeding duration for working mothers [7, 18, 25]. Hence, government may consider a longer paid maternity leave, guarantee frequent and flexible breaks at work, and encourage the provision of an independent breastfeeding room with a refrigerator at the workplaces. In Jiaxing, women can now have a paid maternity leave of 128 days for vaginal delivery and 143 days for cesarean delivery.

### Limitations

This study has some limitations. First, due to the nature of cross-sectional design, we cannot establish causal relationships between EBF and associated factors. Second, this study only enrolled the mothers within our maternal health WeChat groups, so the sample would be underrepresented. It is possible that women who joined in the WeChat groups had stronger intention to breastfeed exclusively than those outside groups. Further study, such as prospective cohort study, is therefore needed to explore the effect of WeChat groups on breastfeeding and women’s perceptions of WeChat groups by comparing women within WeChat groups with those outside. Such a study should also use the WHO definition of EBF in order to be comparable with other studies. Third, the response rate was estimated to be about 75.3%. It is possible that mothers who did not practice EBF were less willing to respond than those who did, which may lead to overestimation of the EBF rate. Fourth, because the mothers completed the questionnaire six months after their delivery, the recall bias could not be avoided. Also the self-report nature of the study may cause reporting bias. Finally, the questionnaire was designed to be relatively simple in order to increase the participation rate. There were some important factors that failed to be measured, such as mothers’ intention and attitude to breastfeeding, the time when mothers introduce complementary foods and stop breastfeeding, supports of husbands and families, etc. These variables have previously been reported as the factors affecting EBF [7, 25] and may provide more information for future breastfeeding education and interventions.

## Conclusions

In summary, this preliminary study shows that 71.3% of mothers within our maternal health WeChat groups exclusively breastfed their infants up to six months, but further comparative study is needed to confirm the effect of WeChat groups on breastfeeding. On the other hand, the perceived insufficient breast milk and work related factors are still the main barriers to EBF up to six months in our study setting. Therefore, future health care should provide more adequate breastfeeding education, especially concerning insufficient breast milk, and promote the provision of a breastfeeding-friendly work environment for working mothers.

## Data Availability

The datasets used and/or analysed during the current study are available from the corresponding author on reasonable request.

## Abbreviations

AOR: Adjusted odds ratio
CI: Confidence interval
EBF: Exclusively breastfeeding
OR: Odds ratio
WHO: World Health Organization
